# To Affinity and Beyond: A Personal Reflection on the Design and Discovery of Drugs

**DOI:** 10.3390/molecules27217624

**Published:** 2022-11-07

**Authors:** Darren R. Flower

**Affiliations:** Independent Interdisciplinary Consultant, Oxford, UK; drdarrenrflower@gmail.com or darrenflower@googlemail.com

**Keywords:** drug discovery, computational chemistry, molecular design, pharmaceutical industry, epistemiology

## Abstract

Faced with new and as yet unmet medical need, the stark underperformance of the pharmaceutical discovery process is well described if not perfectly understood. Driven primarily by profit rather than societal need, the search for new pharmaceutical products—small molecule drugs, biologicals, and vaccines—is neither properly funded nor sufficiently systematic. Many innovative approaches remain significantly underused and severely underappreciated, while dominant methodologies are replete with problems and limitations. Design is a component of drug discovery that is much discussed but seldom realised. In and of itself, technical innovation alone is unlikely to fulfil all the possibilities of drug discovery if the necessary underlying infrastructure remains unaltered. A fundamental revision in attitudes, with greater reliance on design powered by computational approaches, as well as a move away from the commercial imperative, is thus essential to capitalise fully on the potential of pharmaceutical intervention in healthcare.

## 1. Introduction

Human medical need remains, despite humanity’s best efforts; cures for many diseases are persistently elusive and extant strategies are often deeply unsatisfactory. While overall treatment does progress, it is typically through many incremental steps, each with a severely circumscribed effect. Old diseases remain, and new diseases continue to emerge. Until the 20th century, with the dramatic improvement seen in the span of human life, and excepting the contingent and sporadic effects of famine, war, and natural disasters, infectious disease was probably the greatest and most persistent cause of human mortality and morbidity. Today things are very different. In developed nations, and increasingly worldwide, plenty and even affluence have displaced want. There are, it is said, more people alive now who are clinically obese than malnourished. Likewise, the predominant disease burden has switched to so-called “civilisation” diseases and the diseases of old age. Unmet medical need is constant and pressing yet evolving and fluid. The need for new medicines remains as strong if not stronger than ever. Infectious disease and antibiotic resistance apart, humanity still has to contend with cancer, and on a massive scale, as well as the diseases of old age, including dementia and other neurodegenerative maladies. To address unmet need properly requires a healthcare milieu that both delivers the best treatments currently available and seeks new therapies and prophylaxis as an urgent priority. In progressive and enlightened societies, this is the responsibility of wider society, and it should certainly not be the sole province of commerce and big business.

Among other medical interventions, such as surgery and psychological support, the effect of pharmacological medicines has been profound. Originally, and for thousands of years, the only source of drugs was nature—typically but not exclusively extracted from plants as part of complex mixtures—but the rise of synthetic organic chemistry later ushered in an era where drugs and medicines could be created de novo. The Pharmaceutical Industry is the principal engine of drug discovery. The discovery of new medicines—traditionally small molecule drugs, but now expanded to include inter alia biological drugs such as proteins and monoclonal antibodies, vaccines, and medical devices—is in the main orchestrated by commercial interests rather than solely by unmet medical need. It is the profit to be gained from treating chronic and acute disease that guides pharmaceutical companies, not a humanitarian desire to save and improve lives. In this, however, their motives and actions are no better but no worse any other business. Their faults and flaws are common to the rest of commerce and capitalism and are not, as some seem to imagine, unique to Big Pharma. The wider pharmaceutical market is for example happy to medicalise financially remunerative conditions that are not in need of medicalisation while leaving alone rare diseases or those experienced by disadvantaged sectors of society or in impoverished parts of the world alone.

**Figure 1:**
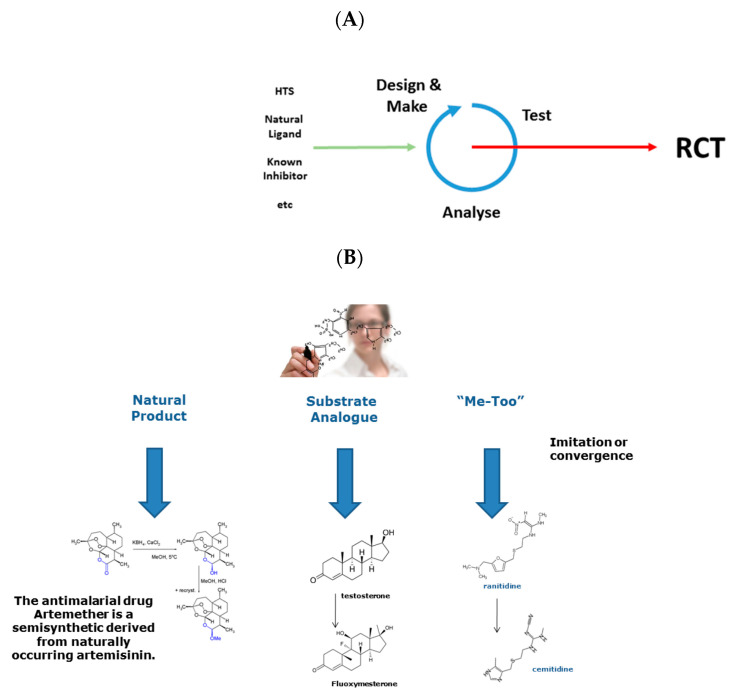
(**A**) the drug discovery paradigm; (**B**) synthetic approaches.

Drug Design is an activity that often forms part of the Drug Discovery process. See Figure 1A. I would be loath to put this proposition more strongly. Not because design never features in the discovery of drugs but because its relative importance is typically overstated, sometimes massively so. I say this not as an opponent of design but as a fierce proponent. Many—perhaps even most—drugs have not been designed, at least not in a complete sense. Many drugs are still derived from naturally occurring substances or other “easy” starting points and have only undergone limited structural modification to yield medically and commercially viable pharmacological agents. See Figure 1B.

Moreover, design per se is not as obvious a concept as it seems. How best to define it? As with most words, definitions vary and any definition is typically heavily context-dependent. Many definitions are available and include: “to create, fashion, or construct according to a plan”, “to conceive and plan to a purpose”, and “to devise or contrive intentionally”. Thus, design as applied on the microscopic scale, the world of molecules, is somewhat different to the design of macroscopic objects, such as tables and chairs. It shares greater similarity to the design of racing cars or bridges, but here the relationship between structure and function is more clearly intuitive, better understood technically, and more intimately constrained. A drug interacts with a supremely complex and still largely unexplored environment, the human body, with its 10,000 s of diverse components from proteins and other biological macromolecules, through cells and tissues, to whole organs. Design—if design it is—needs to operate on many different length scales and time scales. There is no more difficult and complex a design task than knowingly creating a new drug.

In this context, design, as a concept, has wide currency, but the design of molecules as practiced by medicinal chemists is rather more complex and nebulous than the principles of design as applied macroscopically. The kinds of sentiments that are propagated about designing drugs and the sorts of discussion still had around the subject are often no different to the attitudes espoused 20 or 30 years ago. Medicinal chemists still struggle to shake off the narrowness of their focus on making rather than designing molecules. While their ascendancy with the discipline is not as universal as it once was, it remains strong. Design as realised in drug discovery is often a blind process, with only a vague goal in view. Long ago, when affinity or efficacy were the only game in town, the objective seemed clear. In the era of low hanging fruit, there was an abundance of highly “druggable” receptors—GPCRs and the like—that afforded Big Pharma with easy pickings and correspondingly high profits. As these different targets were picked off, and regulatory restrictions tightened, the need to explore a burgeoning diversity of less tractable protein and nucleic acid targets generated in turn an expanding plethora of ever more complex strategies for drug discovery.

In the context of Drug Discovery, we only know in very general terms the nature of what we seek. Even in the era of structure based drug design, we need to begin somewhere, make small quasi-random changes to our starting point, until we ultimately chance upon our goal. With chance being the operative word. Medicinal chemists have at their disposal a limited—if ever growing—repertoire of synthetic transformations they can apply to make stable and synthesisable analogues of molecules. This is a fundamental and ineluctable constraint on design, yet these constraints have become dominant, trouncing other concerns. This blind search is not design, at least not as design is known it in other areas. Even when successful, drug discovery typically takes a very long time and uses much resource. This is problematic enough when the objective is relatively straightforward, but when we add to affinity or efficacy the need for balanced ADME and toxicological properties, amongst others, the process of drug design becomes a daunting technical challenge. Thus, drug design long ago became a multifactorial and multidimensional process where a sizeable set of target-specific constraints are in need of satisfaction.

To solve these dilemmas, Medicinal Chemists have developed a range of rules-of-thumb and other heuristics [1,2]. Many are simple, and, as far as they go, useful. However, among the useful maxims are many shibboleths. People have, for example, and at various points, come to believe in Ligand Efficiency and other metrics that violate among other things the laws of thermodynamics. Other rules, most notably Lipinski’s Rule-of-Five and its various extensions, revisions, and refinements, meant to characterise so-called “drugness”, “drugability” or “drug-likeness”, are transcended by so many active and efficacious compounds as to be more constraining and limiting than useful. Similarly, others have pushed drugs beyond the boundaries of the reduced organic subset to include Boron (Bortezomib, Bavaborole, and Crisaborole for example) and inorganic and organometallic compounds (most famously cisplantin), but these remain—as are so many things—chronically underexplored, likewise for drugs whose targets are sites on chromosomal DNA. These are just a few of the many problems and outstanding issues.

Alternatives to the dominant mantra are dismissed as impractical delusions, or as childish ideas unwisely spoken in the presence of adults. Yet, if Medicinal Chemistry’s ossified lore were indeed as powerful as many assert, we would surely have found optimal drugs for all receptors & all diseases along ago. The fact we have not and continue to struggle to make progress is testament to the fact that devotion to past success can precludes effectively engaging and creating the future. Nobel Prize-winning physicist Richard Feynman famously wrote, “Science is the Belief in the Ignorance of the Experts”. Meaning that science is in constant flux, and belongs to the unknown, or rather the yet-to-be-discovered, future. Expertise by contrast is based on the slow accumulation of valid and validated knowledge, with all the certainty, yet inherent obsolescence, that implies. In this context, the law, as laid down by medicinal chemists, is more expertise than science, only lacking most of the certainty. Things are changing, but like most things in life, far too slowly. In what follows, we explore just a few of the problems that confront Drug Discovery and hint at how some of these might be solved, allowing the discipline to move forward for the benefit of all humanity.

## 2. The Cost of Drugs, the Efficiency of Drug Discovery, and Past Success

In decades past, the obvious failings of extant drug discovery have been mitigated in part by the combination of outrageous good fortune and the idiosyncratic brilliance of isolated research teams. The entropy inherent in the discovery process, often summarised under the catchall term “attrition”, is the defining hallmark of drug development. Costs remain high, driven primarily by the expense of failure. Recent analyses cite wildly different estimates for the cost of newly marketed drugs, yet concur on the scale. Masi et al., 2016 [3] compared 106 NCEs from 10 companies. The average cost per approved drug was estimated at $1.4 billion or, when all other factors are taken into account, $2.9 billion. Compared to previous results from the same team [4,5] costs rose per annum 8.5% above general price inflation. Estimates from more nuanced studies are only marginally more cheery. For example, Prasad & Mailankody [6] found the cost of developing a new cancer drug was $0.7 billion, with post-approval returns averaging $1.7 billion. Jayasundara et al. [7] found the cost of a new orphan drug was $0.2 billion and $0.3 billion for a new non-orphan drug. These typify both the extraordinary expense involved and the context-dependent nature of the analysis. Deeper scrutiny of these and other such studies does little to lessen this impression.

Failure is endemic in drug discovery, and success is rare. This at least is true. Scarcely 1 in 12 drug candidates actually reach the market. Drugs that enter phase II & III clinical trials should possess both activity and safety, as they must, yet they will still have a greater than 90% chance of failure, due predominately to unexpected human side effects or insufficient patient efficacy [4,5]. The difficulty of drug discovery and the ever-escalating costs of bringing a new drug to market has likewise contributed to a reduction in the productivity of the pharmaceutical industry as the engine of drug discovery. Historically, the number of new approvals shows essentially no trend, while financial expenditure on Pharma R&D has risen dramatically [8]. They also find that while research effort rises nine fold, research productivity falls 11 fold by 2007 before rising recently to an overall decline of fivefold by 2014. Research effort rises 6.0% per year, while productivity falls by 3.5% annually. For cancer, productivity of drug research rises until the mid-1980s, before falling. Between 1975 and 2006, cancer research productivity declines 4.8 fold based on clinical trials to approvals.

A perceived and widely acknowledged aspect of the systemic failure of innovation in drug discovery is typified by so-called “me-too” drugs, and their brief discussion is instructive. See Figure 1B. While the long-held assumption was that these drugs are patent-breaking “copies” of already successful drugs, deeper analysis suggests that most arise via a process of convergent evolution seeking to drug the same receptor or disease state [9,10]. Here, structures of me-too drugs originate from different starting points but become increasingly similar as they are constrained both by the limited diversity of synthetic transformations available to medicinal chemists and by the strictures imposed by receptor structure and pharmacokinetics. A me-too drug is related in structure to a first-in-class compound, belongs to the same therapeutic class, is licensed for the same or very similar therapeutic uses, yet differs nominally at least in its pharmacological specificity action, and in its drug–drug interactions and adverse reactions.

Apart from helping during drug shortages, the advantages of a me-too drug may include improved target specificity, reduced off-target activity, fewer drug–drug interactions, some enhanced patient benefit, and augmented drug delivery and pharmacokinetics properties. Importantly, earlier compounds may be as effective as later ones, or more so [11]. Some me-too-heavy drug classes, such as tricyclic antidepressants, contain compounds with similar structures, and which typically offer few if any innovative features, yet are still heavily prescribed. By contrast, me-too β-blockers exhibit considerably more structural diversity. Although many me-too drugs offer no clear benefit over their predecessors, more than 60% of drugs on the WHO’s list of essential medicines are me-too rather than first-in-class drugs.

As a corollary to the travails faced by Big Pharma, there has been a partial opening up of the previously commercially sensitive and thus closed world of drug discovery, with an abundance of open-source and collaborative exercises between pharmaceutical companies and other players such as universities, charities, and publically funded research institutes. We shall not comment further on their success or whether they are more public relation exercises than anything truly substantive.

There are many other factors that contribute to this perceived lack of success evinced by drug discovery; we shall adumbrate just a few. Traditional drug discovery has long relied on animal experiments to evaluate inter alia potency, selectivity, and toxicity. Ethical issues aside, and these are increasingly significant given our burgeoning understanding of animal consciousness and cognition, poorly validated animal experiments often prove to be at best distracting and at worst misleading, providing data that is an unpredictable and inaccurate simulacrum of human disease [12]. After all, one does not dissect a cuckoo clock to find out how a grand piano works, since their mechanisms though they display certain superficial phenotypic similarities are quite distinct. Likewise with animal and human physiology. For example, an adult male BALB/c mouse is approximately 1/4500th the size of an adult human male yet perversely the presumption remains that despite such marked differences in phenotype and genotype, rodent models—probably the most pervasive pre-clinical and toxicology models—provide a seamless surrogate for human disease. Interpreting any animal model is susceptible to the logical fallacy of false analogy, where inferences made likely assume unproven similarities between animal and human disease, leading to embarrassingly erroneous deductions. In the context of drug discovery in particular, the granularity of any validation exercise undertaken to test a model is typically very course, and thus the resulting predictive powers of any such model in terms of absolute drug affinity and other characteristics as well the relative ordering of compounds is typically poor or very poor. Moreover, false positive results are also likely, since animal models typically “reproduce” rather than “replicate” human experimentation, often with a vastly reduced sample size. However, such flawed research continues en masse despite the ready availability of eminently viable and equally reliable alternatives: in vitro approaches, such as human organoids, as well as computational prediction.

In assessing the outcome of drug discovery, we are often dealing with only relatively marginal differences in terms of patient-centric performance. There are exceptions, however, as there are with everything. A recent pertinent example is Dostarlimab and its use in rectal cancer [13]. Other recent innovations, touted as revolutionary game changing advances, including immunotherapy, such as CART-T therapy, genome-informed personalised medicine, and CISPR gene editing, also offer remarkable results in particular cases. However, medicine as a whole and the pharmaceutical industry in particular has been beguiled before. Anti-sense, the human genome project, combinatorial chemistry and parallel synthesis, robot chemistry, and High Throughput Screening (HTS) have all been touted as panaceas that would at a stroke solve the problem of human illness or reduce the stultifying challenges of drug discovery to a wholly automatic and automated process. Even molecular modelling—once—had its time in the sun. HTS in particular seeks to replace “thought with action”, and people once seemed to believe that simply by screening enough compounds, a perfect drug with perfect affinity and selectivity and a perfect pharmacokinetic profile would emerge. How naïve such aspirations seem in hindsight.

The ultimate validation of a drug or vaccine’s efficacy and safety is the double-blind Randomized Controlled Trial, or RCT. Despite their universality, RCTs are nonetheless replete with problems. Apart from being organisationally complex and thus extremely expensive, so-called clinical trials suffer innate systemic problems that call into question their gold standard status. The specific problems of RCTs are but a component or rather an instantiation of a much wider and more fundamental issue within science, that of reproducibility [14]. This is the ability of subsequent experiments or data gathering exercises to replicate with accuracy the findings of previous studies, and thereby help triangulate knowledge and validate prior work. The current perception is that there is a crisis in our ability to replicate or reproduce the results of many scientific studies. In turn, this destabilizes the credibility of theories built on such knowledge, calling into question societal trust in accumulated scientific knowledge. This ongoing methodological and existential crisis is thus undermining faith in the scientific endeavour as a whole because the need to reproduce, or at least validate, empirically determined results is a fundamental requirement of the scientific method.

Demonstrating the reproducibility of RCTs is a special case, and one of singular importance. The questionable validity of clinical trials and similar epidemiological and statistical data gathering exercises is implicit in the need for meta-analysis, where various stratagems are used to combine prior studies in order to reach more unequivocal conclusions about cause and effect. RCTs are seldom if ever directly reproduced. This is partly because a typical RCT is so expensive, both financially and logistically, to run and partly because science despises negative or confirmatory results. Recently, so-called real-world evidence (RWE) has been used to mimic and evaluate RCTs. The FDA’s landmark RCT-DUPLICATE exercise, which used US commercial and Medicare patient claim data to assess the capacity of RWE to evaluate cardiovascular outcomes of antidiabetic or antiplatelet RCTs, gave very mixed results [15]. Six out ten of the trials evaluated could be emulated well using Real-World Evidence (RWE). Apart from highlighting unsurprising deficiencies in both RCTs and RWE methodologies, this exercise suggested much closer agreement when studying hypertension, VTE, and atrial fibrillation, than for asthma, COPD, osteoporosis, heart failure, and chronic kidney disease.

RCTs are seldom large enough or run for long enough, to evaluate a drug properly, particularly in respect of side effects and adverse drug reactions. RCTs are intended to forestall future thalidomide disasters, yet Vioxx still reached market. RCTs are not routinely properly stratified with respect to gender, age, or ethnicity returning only partial, incomplete, and often tendentious data about drug properties, necessitating long-term pharmacovigilance and potential product recalls. In this context, we will elaborate and rehearse some of the more pressing of contemporary areas of unmet medical need: issues with which drug discovery must engage, both now and in the future.

## 3. Vaccine Discovery, Antibiotic Resistance, and Drug Repurposing

Vaccination is the medical intervention par excellence. Its wide deployment during the last 220 years has significantly reduced (95–97%) the mortality and morbidity from a multitude of often lethal infectious diseases: diphtheria, hepatitis B, measles, meningitis, mumps, pneumonia, rubella, and tetanus [16], as well as completely eradicating smallpox and bringing polio to the very edge of extinction. Modern licensed vaccines are whole organism-based or based on single proteins, as well as carbohydrate epitope-based vaccines. Single protein or so-called subunit vaccines are prime targets for vaccine design and reverse vaccinology. Yet, the discovery and development of new vaccines remains reliant on a variety of antiquated and outmoded processes and techniques.

To counter antibiotic resistance, new and effective antibiotics are desperately needed. Yet, the response from the Pharmaceutical Industry has and continues to be less than optimal. This is partly for a number of technical reasons and partly because of the commercial strategies that drive the behaviour companies, which sees other areas of medical need as having a much more satisfactory capacity for remuneration. Many strategies have been suggested to foster antibiotic development [17], but with poor or at best very mixed results. The solution to this dilemma is obvious, but deeply unpalatable: state-funded drug discovery. Only by bringing back science-led drug discovery under public control, can we hope to address the full diversity of societal needs, in particular the discovery of new antibiotics, fully and completely. The issue is compounded by the reluctance of Big Pharma to engage properly with modern design strategies and particularly computational approaches in the discovery of new antibiotics.

**Figure 2:**
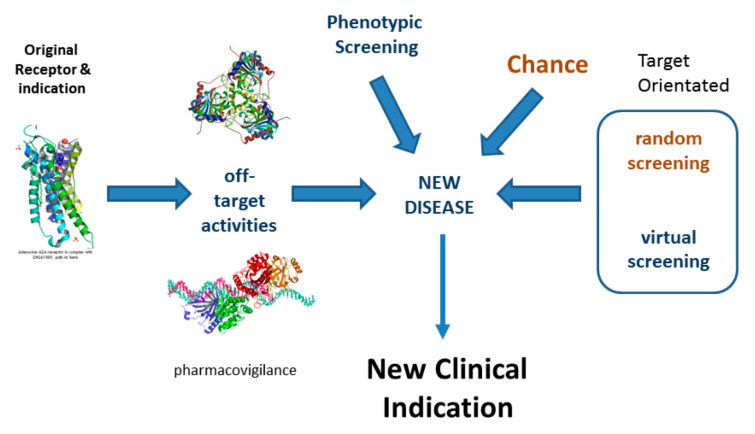
Drug repositioning.

Drug Repurposing is an area of translational biology that identifies novel therapeutically useful indications for marketed drugs by identifying new, disease-relevant biological activities. See Figure 2. Compounds that have been successfully evaluated for safety in Phase I clinical trials but proved unsuccessful for efficacy reasons in Phase II or Phase III trials may also be repurposed. Successful examples of such repositioning abound [18]. Most, maybe all, drugs have significant off-target activity, so drug repurposing has enormous and largely unexploited potential for the identification of safe, novel, well-tested medicines.

Drug Repurposing can also be employed as a strategy for antibiotic discovery. Compounds are sought that inhibit any of the genes identified as essential for bacterial viability in antibiotic resistance bacterial pathogens. The problem is compounded by the number and the cost in terms of both computational time and purchase price of available protein ligand docking software. This is further complicated by the individual restrictions of available docking programs. Some only work well under certain operating systems, while others offer GUI platforms that are compromised in terms of running time, and some do not offer generalised chemical databases. To undertake repurposing for antibiotic discovery requires estimating the highest scoring protein ligand pairs evaluated over hundreds of protein targets tested against marketed drugs and late-stage clinical trial dropouts. The so-called essential genes for many well-studied antibiotic resistant bacteria are known and their corresponding structures have been determined experimentally or can be modelled accurately. This typically yields 300 or 400 protein target structures. The structures of licensed drugs and candidate drugs that have been through phase I clinical evaluation and thus safe, but failed efficacy in phase I or phase II, are also available in large numbers, from databases such as DrugBank (https://go.drugbank.com/, accessed on 20 October 2022) and diverse online repositories. New consensus scoring mechanisms consistently outperform both individual docking platform-based prediction and the most advanced scoring methods currently available. High scoring pairs identified from their consensus scores can then be evaluated properly using a potent combination of in silico and in vitro methods. Assays can measure inter alia bacterial death and the inhibition of growth. Suitable outcomes might include establishing the antimicrobial effectiveness of candidate compounds identified in terms of the minimum inhibitory concentration (MIC) required to inhibit MRSA. Furthermore, time-kill data to establish the kinetics of activity and duration of effectiveness and finally and cross-referencing the characteristics of any challenge panel with the MIC of candidate drugs to establish the scope of effectiveness. Approaches such as this are expected to revolutionise the way docking is presently performed and allow a better, more accessible open sourced approach to computational biochemistry.

## 4. Computational Drug Discovery: AI and Beyond

For a long time, computational approaches to drug and vaccine discovery have remained impoverished Cinderella disciplines starved of attention, funding, and respect. This is despite sufficient evidence to the contrary. To quote the French-American poet and philosopher Anna-Marie le Charas, “Scepticism is healthy, but obstinacy in the face of compelling evidence is not”. Rather like the response to climate science, had computational chemistry been taken seriously and pursued logically, we would not find ourselves in the current mess. Rather than the orchestrated disinformation campaign waged by corporate climate change deniers, computational drug and vaccine discovery has been opposed by the innate complacency of those who feel their hegemony will be supplanted by embracing alternative paradigms. Based on legacy data and properly designed in vitro models, the proper use of in silico methods can help attempts to address the ethically driven need to replace, reduce, and refine the use of expensive, time-consuming, and often highly deceptive, animal experiments, as well as helping eliminate unnecessary clinical trials. Computational models will help the discovery of new antibiotics, kick-start a repurposing revolution that uses automated protein docking to identify testable and already fully safety-tested candidate ligands systematically for all human protein drug targets, and, in its immunoinformatics manifestation, power the design of novel vaccines [19,20,21,22,23].

Due to the perceived success of so-called Artificial Intelligence or AI, and particularly as applied to the field of drug design and discovery, the regressive attitude to computational methods adumbrated above is in the process of being rethought. In this regard, several factors that have converged in AI’s favour. The performance of AI, particularly in translation and image manipulation, has improved to the extent that its results are visible in our everyday digital lives, prompting fears about the supplanting of humanity by nebulous and nefarious AI. Likewise, in science, advances such as DeepMind’s AlphaFold [24], & its academic equivalents such as DeepFold [25] and ColabFold [26], have been heralded as solving the decades-old Protein Folding Problem. More recently, the results of AlphaFold have been made available for the human genome, and more recently for upwards of 200 million protein sequences. If one looks at these for proteins critically, many seem no better than what was already easily predictable using more conventional computational approaches, such as homology modelling. There has also been a widespread application of AI, and generative networks in particular, to drug discovery and design [27,28,29], helping to systematise the synthetic strategies used successfully by medicinal chemists.

Despite a widespread and general acknowledgement of the dramatic improvement in the performance of artificial intelligence during the last decade, AI, with the greatest of possible respect to all those involved, remains as much hype as it does substance. At a fundamental level, AI is what it has always has been, and there is no real difference, fundamentally speaking, to what was achieved 5, 10, or even 20 years ago. There has not been a single, seismic step change in available AI on a technical level; rather it results from greater investment; larger, faster computers and massively more data storage; and the wider deployment of new and established algorithms. At an operational level, AI and machine learning need three things. First, a powerful induction algorithm able to create robust models. We, like others, long ago demonstrated that in the presence of a well-defined problem and sufficient high quality data, existing algorithms were more than sufficient [30,31]. Second, an effective internal representation of data amendable to efficient algorithmic processing. Third, and most importantly, Artificial Intelligence methods are hungry for more and better data, requiring sufficient amounts of data that are also of sufficient quality to allow useful models to be constructed. This is borne out by methods such as DULL-E (https://openai.com/blog/dall-e, accessed on 20 October 2022) and AlphaFold, which are successful primarily because of the mass of data now available to them. Unfortunately, for drug discovery at least, a sufficient amount of quality data usually far exceeds the actual amount of data that is currently available and accessible.

The data needed by computational models and artificial intelligence techniques is highly compromised. Typically, such data is generated by experiments not designed or intended to produce data useful for creating viable and robust computational models. The use of such data in modelling exercises is thus incidental to its original function and thus such use is as a by-product. Were thought processes inverted and the data need by computational science designed and generated explicitly to fulfil the needs of computational models, the data available would necessarily be far more complete, better structured, and with fewer glaring omissions and lacuna, as is often the case with the haphazard and sporadic legacy data currently available. Computational science and AI algorithms need more than just the sweepings from the experimentalist’s table, instead they need large-scale, carefully designed, thorough, and systemic experimental data gathering that only bespoke assays can generate, from which a new generation of more accurate and robust prediction algorithms should spring.

By combining modelling techniques at various length and time scales—dynamic molecular and quantum mechanical models of binding events, Brownian and hydrodynamic models of collisional encounters, course grained models of mesoscale events and structures, with higher scale empirical modelling of compartments and organs—we can hope to model effectively whole organisms, predominantly individual patients with defined genetics. In this way, we can reproduce and perhaps even ultimately replace highly expensive clinical trials with large cohorts of distinct patient models able to evaluate effectively drug efficacy in silico.

## 5. Discussion

Hitherto, drug discovery has progressed through a haphazard process of serendipity driven by the hunt for highly remunerative low hanging fruit [32]. This is simply not sufficient to exploit fully the potential of drugs and vaccines as medical interventions. Nor does conventional Drug Discovery have, as a corporate endeavour, any clearly articulated sense of being systematic. As the ancient Greek Philosopher Hesiod is thought to have said, “It is best to do things systematically, since we are only human, and disorder is our worst enemy”. The Human Genome Project, and all that devolves from it, has shown us, to a first approximation, the protein components comprising the human patient, or at least the overwhelming majority thereof. An obvious objective would then be to find potent, selective inhibitors and ligands for the majority of these target enzymes and receptors. If medicinal chemists—whether working collaboratively with other multi-disciplinary drug discovery scientists or on their own—were able to create and/or design drugs, why has this endeavour not been applied systematically and with more success? The coordinated expression of all human proteins that can be expressed and the subsequent development of inhibition or binding assays would then facilitate the systematic discovery of potent selective ligands—and ultimately drugs, medicines that can cure most diseases—for all “drugable” proteins. A grand scientific endeavour indeed, and at least as realisable as constructing CERN, putting humans on the moon, building the international space station, or assembling the James Webb space telescope.

Unfortunately, corporate drug design remains unable to identify new drugs with ease. This question echoes the age-old trope, “if you are so clever, why are not you rich?” If the discipline was as good as some claim, we should really have all, or nearly all, the drugs we need, but clearly, we do not. There is and never has been a royal road to drug discovery. As Tolstoy put it: “All happy families are alike; each unhappy family is unhappy in its own way”. While there are always many ways to construe any particular statement, a common interpretation is that any process can fail in many ways, and as a process becomes larger and more complex, the ways to fail proliferate.

Conventional Drug Design as practiced by Medicinal Chemistry has been described as a blind man searching through a forest. By combining their other senses, it is possible to navigate their way, moving forward and backward between the trees, with some inevitable collisions, but always by small incremental steps. As they cannot see their distant objective, they only know it has been reached when they stumble upon it. Medicinal Chemistry can of course make molecules and the achievements of synthetic organic chemistry—for that is perhaps what a large part of Medicinal Chemist remains—are well known. Synthetic Organic chemistry has reproduced the structures of complex natural products with relative ease. Yet, here and in other settings, the objective and thus the plan was known, was provided. When trying to design molecules able to fit protein targets and work in patients, there is no clear plan, only a vague you-will-know-when-you-find-it mantra; and de novo design, either human or computational, is rarely if ever successful.

To be able to design molecules, we need to understand, fully & completely, the interactions that potent binders make with proteins, as well as potential targets such as DNA [33]. We need to model effectively a putative drug’s physicochemical properties. Thus, combining the precise binding of a drug candidate to its target and to off-target protein sites with its physicochemical properties should allow us to model drug behaviour within a representative sample of diverse potential patients. This requires data from which to build accurate and robust computational and artificial intelligence models, obtained solely for this purpose from designed experiments rather than collated haphazardly at second hand from innumerable assays and experiments intended for other purposes. Currently, neither poorly orchestrated and underfunded public science nor profit-driven commercial science can address this objective.

What then is the alternative? Science-led, fully systematic, state-funded discovery of drugs and vaccines using reliable in vitro models and robust computational chemistry, powered by artificial intelligence and machine learning. Before recourse to human trials, more stringent validation in simulations comprising large cohorts of genome-based computational models of individual and distinct patients, which are able to take explicit account of genetic variation and developmental variability, and better represent the intrinsic biological variation within patient groups displaying different ethnicities, genders, ages, and physical health. Higher quality drugs would need to be validated by fewer yet larger and better-run double-blind clinical trials, looking for real advantages exhibited by tested drugs, rather than the marginal improvements evinced currently by many newly approved NCEs. This in turn should lead to the definition of a reduced pharmacopeia, comprising fewer yet safer drugs with many more properly understood activities and targeted medical indications, making global health-care at once more completely universal, and more efficient and cost-effective.

Is such a desirable state likely or even possible? Possible, yes, but unfortunately not likely. It is always easier to cling to the emotional reassurance of the past than to embrace the daunting strictures imposed by the future. However, perhaps that is too pessimistic and cynical a prognosis. Instead, let us consider the effect that the remarkable hype surrounding AI has had on the management of the Pharmaceutical industry, and indeed on the wider scientific community, creating a sense of hope and a range of new opportunities. However, misplaced this may prove to be, and anyone with a memory, or a feeling for history, will know that the last 100 years have been replete with false dawns and technical breakthroughs that have not lived up to their hype, let us remain positive. Among these opportunities, is the chance for computational chemistry to have the effect that it could and should have had decades ago, allowing true molecular design to flourish [34,35].

## 6. Conclusions and Future Perspective

For evidenced-based Drug Discovery to flourish, it must of needs be based on the principles of design and well-founded computational chemistry not on heuristics. To achieve this, a new generation of accurate and comprehensive predictive models is required, which are able to model drug interactions with their biological environment, both their sites of action and the wider milieu, both fully and completely. Models that can capture activity, the full range of ADME properties, and off-target activities, and thus predict overall efficacy and forecast individual side effects accurately based on personal genome profiles. To do this, we need large-scale de novo generation of bespoke data designed to address the creation of predictive models and not data created by a highly biased, haphazard, and sporadic process of incidental data gathering. Pursuing this goal should provide a much more hopeful and optimistic outlook for drug design within the Drug Discovery process.
